# *Mycobacterium bovis* BCG Interferes with miR-3619-5p Control of Cathepsin S in the Process of Autophagy

**DOI:** 10.3389/fcimb.2016.00027

**Published:** 2016-03-09

**Authors:** Kamlesh Pawar, Jutta Sharbati, Ralf Einspanier, Soroush Sharbati

**Affiliations:** Department of Veterinary Medicine, Institute of Veterinary Biochemistry, Freie Universität BerlinBerlin, Germany

**Keywords:** microRNA, *Mycobacterium bovis* BCG, phagocytosis, Cathepsin S, miR-3619-5p, autophagy

## Abstract

Main survival mechanism of pathogenic mycobacteria is to escape inimical phagolysosomal environment inside the macrophages. Many efforts have been made to unravel the molecular mechanisms behind this process. However, little is known about the involvement of microRNAs (miRNAs) in the regulation of phagolysosomal biosynthesis and maturation. Based on a bottom up approach, we searched for miRNAs that were involved in phagolysosomal processing events in the course of mycobacterial infection of macrophages. After infecting THP-1 derived macrophages with viable and heat killed *Mycobacterium bovis* BCG (BCG), early time points were identified after co-localization studies of the phagosomal marker protein LAMP1 and BCG. Differences in LAMP1 localization on the phagosomes of both groups were observed at 30 min and 4 h. After *in silico* based pre-selection of miRNAs, expression analysis at the identified time points revealed down-regulation of three miRNAs: miR-3619-5p, miR-637, and miR-324-3p. Consequently, most likely targets were predicted that were supposed to be mutually regulated by these three studied miRNAs. The lysosomal cysteine protease Cathepsin S (CTSS) and Rab11 family-interacting protein 4 (RAB11FIP4) were up-regulated and were considered to be connected to lysosomal trafficking and autophagy. Interaction studies verified the regulation of CTSS by miR-3619-5p. Down-regulation of CTSS by ectopic miR-3619-5p as well as its specific knockdown by siRNA affected the process of autophagy in THP-1 derived macrophages.

## Introduction

Mycobacteria consist of both highly pathogenic species e.g., *Mycobacterium tuberculosis* and opportunist species such as *M. avium*. Once entered into the host cell, most of the bacteria are trafficked into a series of increasingly acidified membrane-bound structures that lead to eradication of bacteria, by a process called phagosomal maturation in macrophages. However, pathogenic mycobacteria have the ability to survive in this harsh environment. Phagosomal maturation is divided into three different stages, which are early, intermediate and late that culminate with the formation of phagolysosomes (Flannagan et al., 2009). The phagosome acquires the proteins Rab5 and EEA on its membrane during early maturation stages, whereas LAMP1 and Rab7 are present on late maturation stages (Gorvel et al., 1991; Vieira et al., 2003; Flannagan et al., 2009). To understand mycobacterial survival strategies, a number of different proteins (e.g., Rab5, Rab7, EEA1, hVPS34 etc.) that are involved in phagosomal maturation have been studied on the surface of mycobacteria containing phagosomes (Gorvel et al., 1991; Christoforidis et al., 1999; Bucci et al., 2000; Fratti et al., 2001; Vieira et al., 2003). Studies have focused on how mycobacteria change phagosomal maturation to avoid its eradication (Vergne et al., 2004). Mycobacteria manipulate acidic environment of phagosomes by affecting several mechanisms. It has been shown that phagosomes containing *M. tuberculosis* and *Mycobacterium bovis* BCG (BCG) interact normally with early endosomes but fail to fuse with late endosomes and lysosomes (Sun et al., 2007). Moreover, it has been documented that mycobacteria interact with endosomal-lysosomal pathways (Clemens and Horwitz, 1995) but prevent the acidification of phagosomes to survive and multiply inside the host cells (Sturgill-Koszycki et al., 1994; Xu et al., 1994). Relative to phagosomes containing latex beads, BCG phagosomes have been shown to be depleted in LAMP2, NPC1, flotillin-1, vATPase, and syntaxin 3 (Lee et al., 2010). Beside these differences, novel proteins have been detected on BCG phagosomes but not on latex bead containing ones, which include CD44, ICAM-1, FAM3C, RALA/RALB, STIP1, EPB41L3, SEPT7, IQGAP1, Rab-6A, erlin-2, and Tpd52l2 (Lee et al., 2010). However, there are still gaps in understanding how mycobacteria interfere with endosomal trafficking and its regulation.

Apart from several other diseases, involvement of miRNAs in the immune response to pathogens provided allusion of their role in mycobacterial pathogenesis. When human macrophages were infected with virulent *M. tuberculosis*, high miR-125b and low miR-155 expression was observed compared to infection with the non-virulent *M. smegmatis* (Tili et al., 2007; Rajaram et al., 2011). BCG suppressed IFN-γ production by down-regulating miR-29 expression in IFN-γ-producing natural killer cells (Ma et al., 2011). An integrated miRNA-mRNA analysis of infected human macrophages provided insights into the cumulative impact of miRNA regulation during *M. avium* infection of human macrophages (Sharbati et al., 2011). BCG triggers TLR2-dependent miR-155 expression in macrophages, which involves signaling cross talk among PI3K, PKCδ, and MAPKs, and recruitment of NF-κB and c-ETS to miR-155 promoter. This BCG-driven miR-155 expression acts as a crucial regulator of cell fate decisions of infected macrophages (Ghorpade et al., 2012). Intracellular survival of *M. tuberculosis* was decreased when mouse macrophages were transfected with miR-155 (Kumar et al., 2012). The reason might be because miR-155 promotes autophagy to eliminate intracellular mycobacteria by targeting RHEB, which is a negative regulator of autophagy (Wang et al., 2013).

Autophagy is a process where cells remove or recycle damaged and unwanted cytosolic components to provide amino acids during starvation. It also helps disposing microorganisms. An important marker for autophagy is LC3. After lipidation of the cytosolic form (LC3-I), LC3-II is recruited to autophagic membranes (Klionsky et al., 2007). In case of BCG infection, induction of autophagy by IFN-γ leads to engulfment of bacteria in autophagosomes and reduced viability in macrophages (Gutierrez et al., 2004). Autophagy, phagocytosis, and endocytosis are interconnected cellular processes as phagosomal membranes have been shown to recruit LC3 in a TLR-dependent fashion (Sanjuan et al., 2007). TLR-induced association of autophagic components promotes phagosome–lysosome fusion. So, TLR signaling enhances phagosomal maturation and thereby pathogen defense coinciding with autophagy related pathways (Sanjuan et al., 2007). On a different note, few recent studies have suggested that inhibition of the lysosomal cysteine protease Cathepsin S (CTSS) affects autophagy in different cells (Chen et al., 2012; Huang et al., 2013, 2015; Zhang et al., 2014) as observed by enhanced LC3 conversion.

miRNAs are considered to fine tune the regulation of gene expression and are involved in various processes in mycobacterial infection. Finding new miRNAs in mycobacterial infection gave new dimensions to study its pathogenesis, however few have focused on regulation of phagolysosomal processing and autophagy by miRNAs. The present study mainly focused on an approach to find miRNAs in phagolysosomal maturation during infection of THP-1 derived macrophages with viable and heat killed (HK) BCG and provided functional aspects to the regulated RNAs. Beside *in silico* predictions of relevant miRNAs, time points of investigation were identified, based upon presence or absence of LAMP1 as a marker for phogolysosome formation on HK and viable BCG phagosomes. After *in silico* prediction of binding partners, infection dependent dysregulation of predicted miRNAs as well as targets was examined and validated experimentally. In the end, functional relevance of a regulated miRNA was associated with the process of autophagy.

## Materials and methods

### Bacterial cultivation, cell culture, and infection experiments

*M. bovis* BCG (DSMZ No. DSM 43990) was grown as previously described (Sharbati-Tehrani et al., 2004). For microscopy, BCG was fluorescently labeled with 5-(6)-carboxyfluorescein-succinylester (Sigma Aldrich) following an earlier described protocol (Agerer et al., 2004). The human acute monocytic leukemia cell line THP-1 (DSMZ ACC 16) was cultured in suspension using RPMI 1640 (Biochrom AG) supplemented with 10% fetal bovine serum superior (Biochrom AG) and gentamycin (zur Bruegge et al., 2014). Infection experiments were performed as described previously (Pawar et al., 2016).

### LAMP1 detection and determining time points for RNA isolation

For selection of time points for RNA isolation, LAMP1 signal was observed during mycobacterial infection. In brief, differences in LAMP1 signal were observed on phagosome containing viable and HK mycobacteria and time points for RNA isolation were selected based on presence or absence of LAMP1 signal. To coordinate phagocytosis, cell and mycobacterial interaction was synchronized by incubating THP-1 derived macrophages at 4°C for 10 min without mycobacteria then at 4°C with mycobacteria for 10 min (Stewart et al., 2005). Afterwards, cells were incubated at 37°C in a CO_2_ incubator and presence or absence of LAMP1 signal on phagosomal membranes was observed every 15 min after washing and fixing the cells in 4% Roti-Histofix (Carl Roth). Immunostaining was performed as described previously (Sun et al., 2007; Sharbati et al., 2015) using a 1:300 dilution of the antibody LAMP1 [1D4B] (ab25245) Rat mAb (Abcam) together with the secondary antibody Goat pAb to Rat IgG, Alexa Fluor 594 (ab150160) (Abcam) using a 1:300 dilution. Microscopic photographs were taken with an inverted fluorescence microscope DMI6000 B (Leica). Nuclei were counterstained using DAPI (Sigma-Aldrich). Consequently, 15 min, 30 min and 4 h were chosen for RNA isolation. Total RNA was isolated using the miRVana Isolation Kit (Life Technologies). The quality and quantity was controlled as described previously (Sharbati et al., 2010).

### Approach for selecting miRNAs and protein-coding genes

(1) The search for protein-coding genes involved in the pathways: Three pathways [Phagosome (ko04145), Lysosome (ko04142), and Tuberculosis (hsa05152)] from KEGG pathway database (Kanehisa and Goto, 2000; Kanehisa et al., 2014) were searched for protein-coding genes based on their direct involvement in the phagosomal maturation process and were backed up by literature (Supplementary Table 1). (2) miRNAs targeting the selected genes were chosen using miRmap (Vejnar and Zdobnov, 2012) possessing a score of 90 and above (Data Sheet 1). (3) Enriched miRNAs were selected with the help of the Galaxyproject (Blankenberg et al., 2010). Top 10 mostly enriched miRNAs were chosen for the study (Supplementary Table 2). (4) After finding regulated miRNAs, corresponding targets were again searched in miRmap using a stringent score of 99 and above (Data Sheet 2). (5) List of identified targets was loaded on the database for annotation, visualization and integrated discovery (DAVID) (Dennis et al., 2003) and genes were selected based on their involvement in phagosomal maturation related pathways [Lysosome (ko04142), Phagosome (ko04145), Endocytosis (ko04144), Antigen presentation (hsa04612)] from KEGG pathway classification in annotation summary result. Genes were selected for the expression study based on the criteria that at least two mutual miRNAs target one protein-coding gene with a miRmap score of 80 and above (Table 1).

**Table 1:** **miRmap score of miRNAs targeting the genes**.

**Name of gene**	**miR-3619-5p (miRmap score)**	**miR-637 (miRmap score)**	**miR-324-3p (miRmap score)**
GIT1	15.19	13.98	99 and above
**IQSEC3**	**96.56**	**95.37**	**99 and above**
**RAB11FIP1**	**99.35**	**no target**	**99 and above**
RAB11FIP3	47.27	4.20	99 and above
**RAB11FIP4**	**99.82**	**99.97**	**99 and above**
ARRB1	57.01	27.47	99 and above
PIP5K1C	47.84	0.25	99 and above
**PIP4K2B**	**98.34**	**99.38**	**99 and above**
**ZFYVE20**	**90.99**	**No target**	**99 and above**
**EHD3**	**3.52**	**99 and above**	**96.15**
**RAB11FIP4**	**99.82**	**99 and above**	**99.80**
**TRAF6**	**82.54**	**99 and above**	**No target**
GGA1	40.33	99 and above	46.91
**PIP4K2B**	**98.34**	**99 and above**	**99.64**
**ARFGAP2**	**99 and above**	**89.29**	**No target**
**RAB11FIP1**	**99 and above**	**no target**	**99.64**
**RAB11FIP4**	**99 and above**	**99.97**	**99.80**
AP3B1	99 and above	no target	No target
**CTSS**	**99 and above**	**98.32**	**No target**
**GGA3**	**99 and above**	**95.94**	**No target**
PSD3	99 and above	no target	69.50

*Bold fonts indicate “Protein-coding genes” that have at least two mutual miRNA targets with a miRmap score of 80 and above*.

### Quantification of mRNA and miRNA by RT-qPCR

Quantification of mRNA as well as miRNA expression by means of RT-qPCR was performed as described earlier by triplicate measurement of individual samples (Sharbati-Tehrani et al., 2008; Sharbati et al., 2010, 2011). All experiments were performed according to MIQE guidelines (Bustin et al., 2009) and based on the ΔΔCq method (Livak and Schmittgen, 2001) following the protocols described previously (Sharbati et al., 2012; Pawar et al., 2016). For normalization of expression data, the stability of reference RNAs was tested using the geNorm algorithm (Vandesompele et al., 2002) and the geometric means of reference RNAs were calculated for normalization. For normalization of mRNA expression, GAPDH, B2M as well as ACTB were used, which all possessed stable expression. For normalization of miRNA expression, SNORD44 and SNORD52 were used as reference RNAs. The entire set of oligonucleotides used in this study is provided in the Supplementary Table 2. All oligonucleotides were synthesized by Sigma Aldrich.

### RNAi experiments, detection of autophagy, and western blots

THP-1 cells (1 × 10^6^) were transfected using the Nucleofector Technology (Lonza) together with 50 pmol miRNA mimic miR-3619-5p (5′-ucagcaggcaggcuggugcagc-3′), miR-637 (5′-acugggggcuuucgggcucugcgu-3′) or 100 pmol of On-Targetplus human CTSS siRNA (SO-2494920G, Dharmacon) using Kit V (Lonza). After transfection, THP-1 cells were differentiated into macrophages using 10 ng/ml phorbol-12-myristate-13-acetate (PMA, Sigma Aldich) and incubated for 24 h and RNA was isolated to observe expression level of RAB11FIP4 and CTSS. Knockdown of CTSS was evaluated by means of western blot experiments using a 1:500 dilution of rabbit anti-CTSS primary antibody (ab134157, Abcam) together with a 1:10000 dilution of the horseradish peroxidase-conjugated anti-rabbit secondary antibody (Cell Signaling Technology). Experiments were performed three times for statistical analysis. For detection of autophagy after transfection with miR-3619-5p mimics and CTSS siRNA, LC3A/B, and p62 proteins were detected by means of western blotting as described earlier (Pawar et al., 2016). For counting LC3 puncta, at least six randomly selected microscopic photographs of each biological replicate were analyzed using ImageJ 1.48v (Schneider et al., 2012). Fluorescent staining for microscopy and method of analysis for counting LC3 puncta was done as described earlier (Pawar et al., 2016). To validate the autophagy signal produced in transfected experiment, another approach for detection of LC3 signal was used. THP-1 cells were co-transfected with miR-3619-5p and the reporter plasmid pEX-GFP-hLC3-WT (Addgene). Autophagy signal (number of LC3-GFP puncta) produced in above cells was compared with that of THP-1 cells co-transfected with nonsense control and pEX-GFP-hLC3-WT plasmid. Minimum of 15 autophagic cells were counted per biological replicate (*n* = 3) in each group.

### Reporter gene assay

Reporter gene assays were performed as described previously (Sharbati et al., 2011). HeLa cells were transfected using the Nucleofector Technology (Lonza). Nucleofection was performed with 5 × 10^5^ cells per transfection using 2 μg reporter plasmid (pTK-Gluc derivatives, NEB GmbH), 200 ng normalization plasmid (pTK-Cluc, NEB GmbH), and 100 pmol miRNA mimic according to the manufacturer's instructions. In brief, three miR-3619-5p target sites of human CTSS were identified (position 38, 1656, and 2477) and used as a single unit oligonucleotide—NotIhCTSS-ts-sense and XbaIhCTSS-ts-antisense (Supplementary Table 2)—that were hybridized and cloned in pTK-Gluc as described earlier (Hoeke et al., 2013). Endotoxin-free reporter plasmids (pTKGhCTSS) were produced for transfection.

### Statistical analysis

Unpaired *t*-tests were conducted to test significant differences between two treatments. Asterisks in figures summarize *P*-values (^*^P < 0.05). Statistical tests were conducted applying GraphPad Prism version 6.00 for Windows, GraphPad Software, La Jolla California USA, www.graphpad.com.

## Results

### LAMP1 was absent on phagosomes containing viable BCG

**Figure 1:**
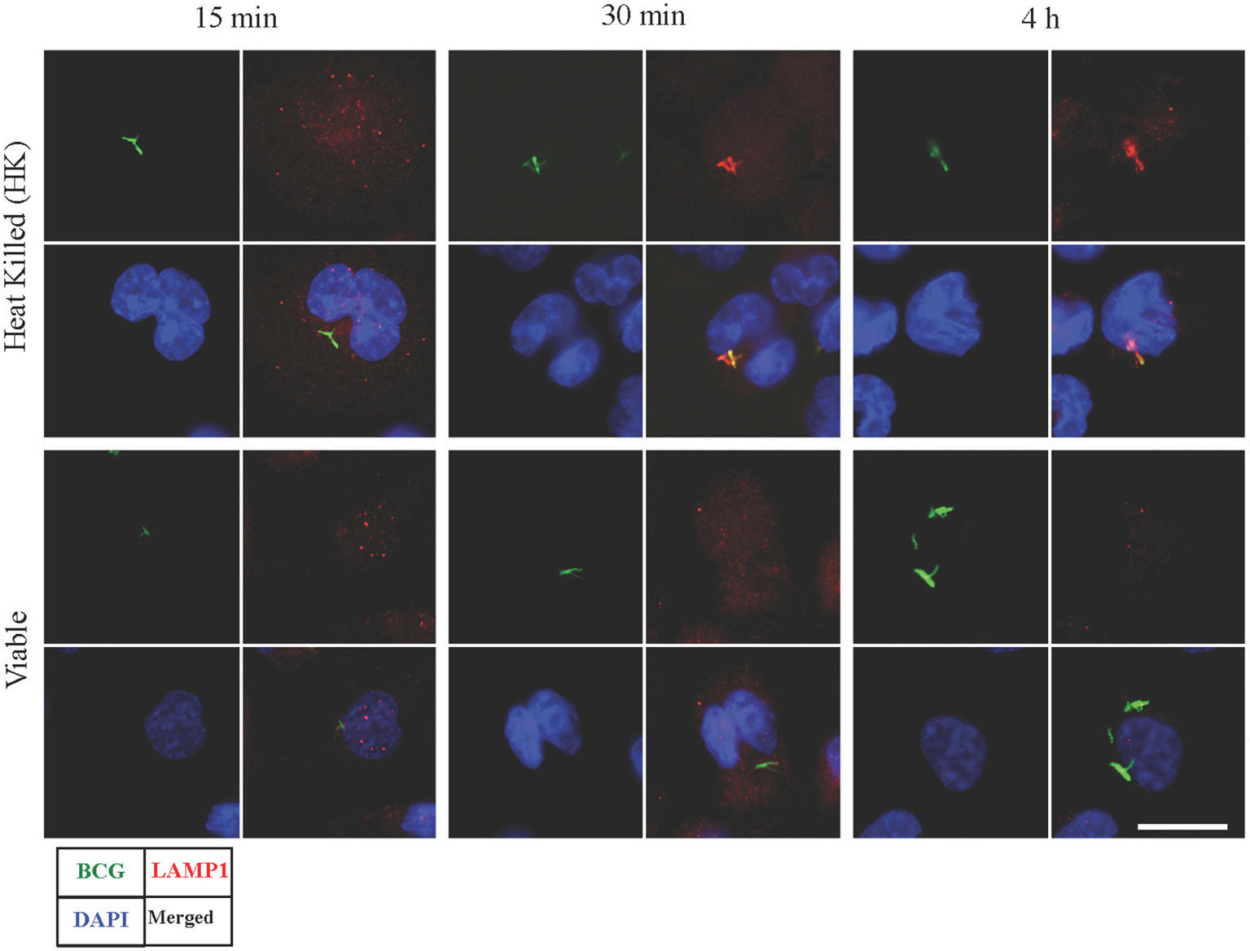
**Detection of LAMP1 signal in macrophages infected with viable and heat killed (HK) ***Mycobacterium bovis*** BCG**. Bacteria were stained with FITC (green fluorescent). At 15 min, no visible LAMP1 signal was observed that are co-localized with BCG in both viable and HK bacterial infection. At 30 min and 4 h, LAMP1 signal was visible in HK samples co-localizing with HK BCG, however no equivalent signal was seen in viable bacterial infection at 30 min and 4 h. Bar measures 25 micron.

Synchronized infection experiments were performed and LAMP1 signal (as a late phagosomal marker) was studied on viable and heat killed (HK) BCG containing phagosomes for up to 4 h post infection. There was no LAMP1 signal observed in initial period of time (at 15 min) in both types of infection (Figure 1). However, the difference in signals was observed after 30 min and onward. At 30 min and 4 h, LAMP1 signal was visible in HK BCG infection co-localizing with BCG and no equivalent signal was seen in viable BCG infection at 30 min and 4 h. Therefore, 30 min and 4 h were chosen representing the time points, where BCG actively manipulate endosomal trafficking. The time point 15 min served as an additional early control.

### Selection of interacting miRNAs and mRNAs by means of a bottom up approach

**Figure 2:**
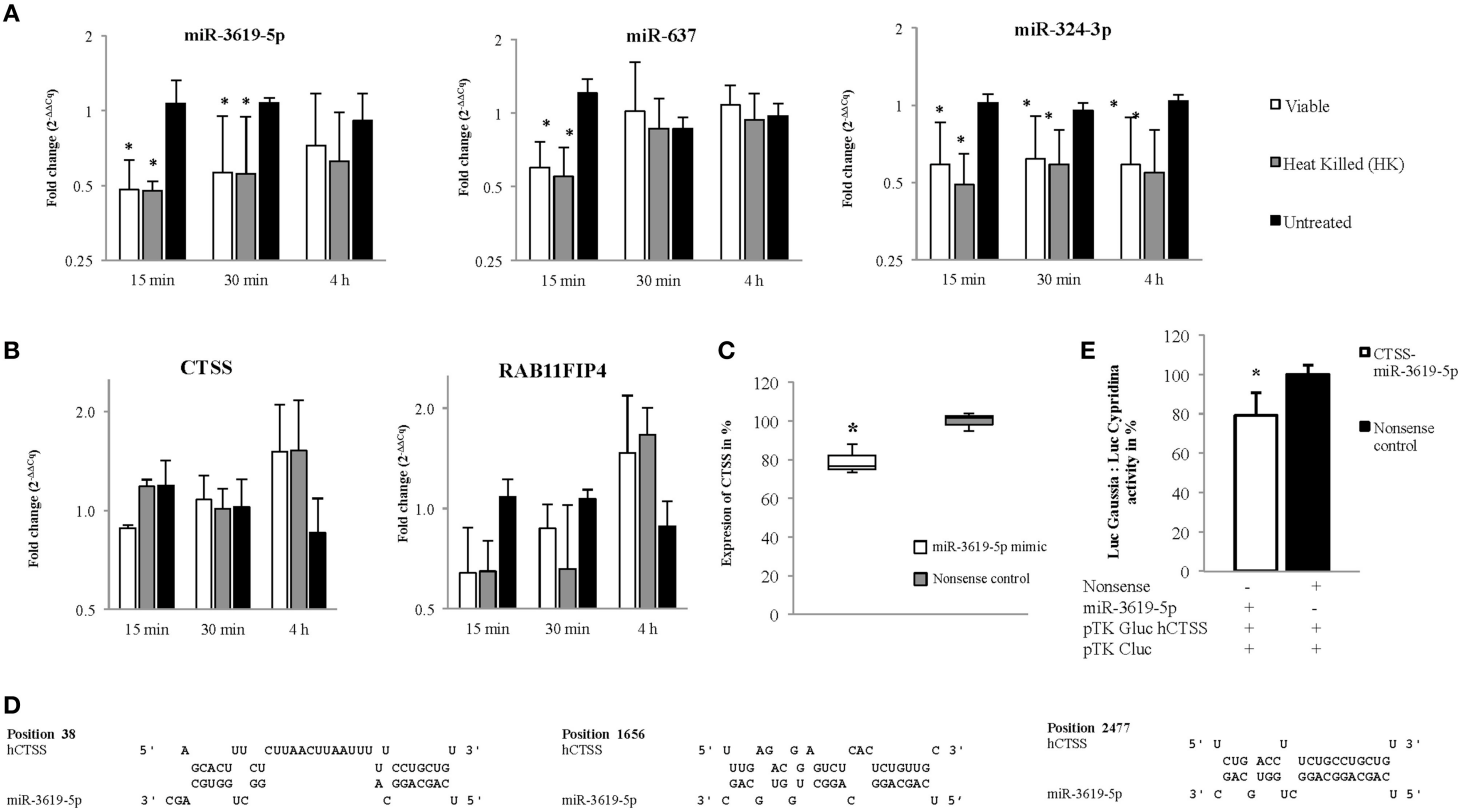
**Expression of miRNAs and their predicted targets at 15, 30 min, and 4 h in viable and heat killed (HK) mycobacterial infection to macrophages. (A)** The miRNAs, miR-3619-5p and miR-324-3p were down-regulated in all time points whereas miR-637 was down-regulated only at initial period of time (15 min) in both viable and HK bacterial infections. **(B)** The predicted targets, CTSS and RAB11FIP4 were up-regulated in later period of time points (4 h) in both viable and HK bacterial infections. **(C)** Expression of CTSS in THP-1 cells was down-regulated after transfection with miR-3619-5p mimics. **(D)** Three different sites were found after screening the 3′ UTR region of CTSS for potential miR-3619-5p binding sites using RNAhybrid. **(E)** The interaction between miR-3619-5p and human CTSS was verified by means of reporter gene assays using HeLa cells. Identified target sites between miR-3619-5p and CTSS were analyzed using RNAhybrid. Relative luciferase activity (Luc _Gaussia_: Luc_Cypridina_) was determined respective to nonsense miRNA mimics. The columns show means of three biological replicates each measured in triplicates while error bars show the standard deviation. Asterisks indicate statistical significance between samples (^*^P < 0.05).

A total of 15 different proteins were selected based on their direct involvement in the phagosomal maturation process as well as on literature search. Selected protein-coding genes are shown in Supplementary Table 1. After searching miRNAs targeting each 15 different genes, lists of identified miRNAs were merged (resulted in a total of 1840 miRNAs) (Data Sheet 1). Out of 1840 miRNAs, few miRNAs were targeting more than one protein-coding gene. For example, miR-3619-5p, miR-4739, and miR-637 were predicted to target 6 different genes, whereas miR-324-3p, miR-4446-5p, miR-4690-5p, miR-4709-3p, miR-5006-5p, miR-654-5p, and miR-761 were supposed to target five different genes. Remaining miRNAs targeted four or less different genes. Most enriched miRNAs (having six and five targets) were considered to have relevant functions for the study. So, top 10 most enriched miRNAs were chosen for infection based expression studies by means of RT-qPCR (Supplementary Table 2). Out of 10, three miRNAs (miR-3619-5p, miR-637, and miR-324-3p) were down-regulated compared to non-infected controls (Figure 2A). Other 4 miRNAs (miR-4446-5p. miR-654-5p, miR-4709-3p, and miR-761) out of 10 were not regulated (Supplementary Figure 1A) and 3 miRNAs (miR-5006-5p, miR-4690-5p, and miR-637) were below the detection limit. miRNAs that were dysregulated, were used for further *in silico* analysis. Again highly potential targets of three dysregulated miRNAs (miR-3619-5p, miR-637, and miR-324-3p) were searched using miRmap with a score of 99 and above (Data Sheet 2). The predicted gene list was searched for protein-coding genes that were supposed to be mutually targeted by at least two miRNAs where the subsequent interactions had a miRmap score of 80 and above (Table 1). This resulted in identification of protein-coding genes (RAB11FIP4, PIP4K2B, RAB11FIP1, ZFYVE20, TRAF6, and CTSS) and their expressions were studied in same RNA samples that were used for miRNA expression studies helping to determine anti-correlated expression of supposed targets (Supplementary Table 2 and Figure 1B). Expression of other mutually targeted protein-coding genes (EHD3, IQSEC3, ARFGAP2, and GGA) was below detection limit.

### miR-3619-5p, miR-637, and miR-324-3p and the proposed targets RAB11FIP4 and CTSS exhibited anti-correlated expressions

MiRNAs bind to respective mRNAs causing inter alia their degradation. In our experiments, down-regulation of three miRNAs miR-3619-5p, miR-637, and miR-324-3p was observed, and two mRNAs RAB11FIP4 and CTSS were up-regulated or at least showed a trend in up-regulation. Expression of miRNAs as well as mRNAs from same RNA samples is given in Figures 2A,B, respectively. Based on miRmap score analysis, all three miRNAs were supposed to target RAB11FIP4, however CTSS was predicted to be targeted by miR-3619-5p and miR-637, respectively. Therefore, we concentrated on studying the mutual targets RAB11FIP4 and CTSS of miR-3619-5p and miR-637. Consequently, transfection experiments with miR-3619-5p and miR-637 mimics were performed and cellular levels of both identified targets were examined. As shown in Figure 2C, only transfection of THP-1 with miR-3619-5p mimics resulted in significantly reduced levels of cellular CTSS and there was no effect of miR-3619-5p on RAB11FIP4 and miR-637 on both CTSS and RAB11FIP4, respectively (Supplementary Figure 2).

### miR-3619-5p targets CTSS

Three different binding sites were found (Figure 2D) after screening the 3′ UTR of CTSS for potential miR-3619-5p interaction using RNAhybrid (Kruger and Rehmsmeier, 2006). Calculated minimal free energies for interactions with miR-3619-5p were −31.2 kcal/mol, −22.1 kcal/mol, and −17.1 kcal/mol, respectively (Figure 2D). Reporter gene assays with identified binding sites in CTSS were performed (Supplementary Table 2). After co-transfection of miR-3619-5p mimic or nonsense controls together with the reporter plasmid, a significant reduction in luciferase activity (Luc_Gaussia_: Luc_Cypridina_) was measured for the interaction (*P* < 0.05, paired *t*-test) compared to nonsense controls (Figure 2E). This down-regulation (20%) of CTSS was comparable to the reduction of CTSS transcript levels in transfected THP-1 cells by miR-3619-5p mimic. This experiment proved the interaction between CTSS binding sites and miR-3619-5p to be specific.

### miR-3619-5p as well as siRNA-mediated knockdowns of CTSS affect the process of autophagy

**Figure 3:**
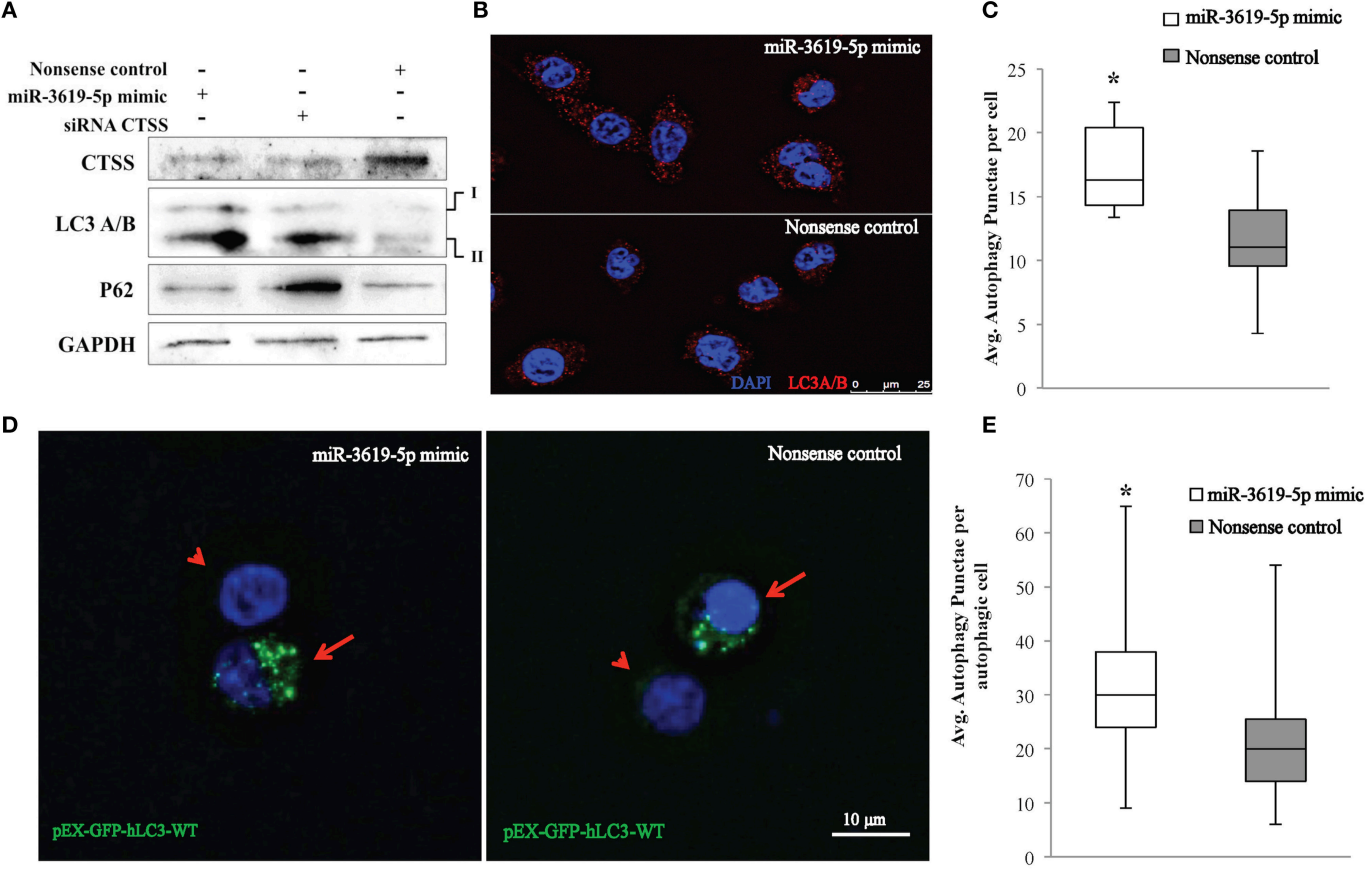
**Detection of autophagy in THP-1 cells transfected with miR-3619-5p mimics. (A)** Expression of CTSS in THP-1 cells was down-regulated after transfection with miR-3619-5p mimics as well as siRNA, which affected the process of autophagy observed by LC3A/B conversion on western blots. **(B)** LC3 autophagy puncta were observed under the microscope, **(C)** and were significantly increased in miR-3619-5p mimics transfected cells compared to nonsense controls as calculated by ImageJ. **(D)** LC3-GFP autophagy puncta were also observed with pEX-GFP-hLC3-WT plasmids. Co-transfecting cells with miR-3619-5p with pEX-GFP-hLC3-WT plasmid produced more autophagy GFP-LC3 puncta than that of co-transfected cells with nonsense control and pEX-GFP-hLC3-WT plasmid as observed under fluorescent microscope. Arrow indicates co-transfected cells and arrowhead indicates non-transfected population. **(E)** Significant differences in number of autophagic GFP-LC3 puncta was observed between two groups counted by ImageJ. The columns show means of three biological replicates each measured in triplicates while error bars show the standard deviation. Asterisks indicate statistical significance between samples (^*^P < 0.05).

Ectopic expression of miR-3619-5p in THP-1 derived macrophages reduced the level of CTSS transcripts (Figure 2C). As mentioned above, CTSS has been connected to autophagy. To test for functional effects of miR-3619-5p mediated CTSS regulation in macrophages; we examined the effect of miR-3619-5p transfection on autophagy. For this purpose, LC3A/B conversion was determined by means of western blots showing clearly increased LC3A/B-II levels after miR-3619-5p transfection compared with nonsense treated controls (Figure 3A). To test if the observed effect is grounded in decreased cellular levels of CTSS, we used specific siRNA, which again enhanced conversion of LC3A/B (Figure 3A). Western blots proved clearly decreased cellular levels of CTSS after both miR-3619-5p and siRNA transfection (Figure 3A). The degradation of p62 is regarded to be a marker for progression of autophagy. Therefore, we examined cellular p62 levels after RNAi. Interestingly, there was no degradation of p62 after miR-3619-5p transfection and p62 even increased after specific knockdown of CTSS (Figure 3A) indicating block of lysosomal processing after CTSS knockdown.

As shown in Figure 3B, the process of autophagy was also examined by immunofluorescence quantification of LC3A/B puncta. Significantly increased LC3 puncta were observed in mimic transfected cells compared to nonsense-transfected cells (Figures 3B,C) as determined by ImageJ. Accumulation of LC3 puncta determined by immunofluorescence after transfection with miR3619-5p was confirmed by co-transfecting THP-1 derived macrophages with the GFP-LC3 reporter plasmid pEX-GFP-hLC3-WT (Tanida et al., 2008). Co-transfection of cells with miR-3619-5p and pEX-GFP-hLC3-WT produced increased number of GFP-LC3 puncta compared to co-transfected cells with nonsense controls and pEX-GFP-hLC3-WT (Figure 3D). Significant difference in number of autophagy LC3 puncta was observed between two groups determined by ImageJ (Figure 3E).

## Discussion

Phagosomal maturation goes along with changes in the protein composition of phagosomal membranes. Lysosomal fusion with phagosomes is an important stage in maturation, which is required for degradation or digestion of foreign particles as well as apoptotic bodies. Gaining LAMP1 protein on phagosomal membrane indicates phagosomal fusion with lysosomes. However, progression of phagosomal maturation fails when pathogenic mycobacterial species are phagocytized. In present investigation, based on knowledge of phagosomal maturation in mycobacterial infection, three different time points were selected, where absence and presence of LAMP1 proteins on phagosomes containing viable and HK BCG was observed. In this study, LAMP1 signal was noticeably present at 30 min, which co-localized with HK BCG. However, same signal was absent in case of viable BCG at 30 min and onwards. This indicates that phagosomes containing viable BCG failed to fuse with lysosomes compared to phagosomes containing HK BCG. As observed previously, wild-type BCG were associated with LAMP1-negative vesicles while BCG with inactivated protein serine/threonine kinase G (ΔpknG) co-localized predominantly with the lysosomal marker LAMP1 (Walburger et al., 2004). It was concluded that the mycobacterial protein serine/threonine kinase G (PknG) in BCG carries out the biological function in blocking lysosomal delivery of mycobacteria (Houben et al., 2009). In present case, heat killing of BCG might have inactivated PknG in BCG leading to fusion of phagosome containing HK BCG with lysosome. However, this finding was not in agreement with some other studies that showed BCG was associated with LAMP1 positive phagosomal compartments (Billeskov et al., 2010; Lee et al., 2010). Different bacterial strains or cell lines might be the cause for these differences. Primary aim of studying phagosomal LAMP1 was to select time points for RNA isolation during mycobacterial infection, which was used in a bottom up approach to select miRNAs that might be involved in the process of phagolysosomal trafficking. It was important to determine the exact time for RNA isolation to increase the chance for observing distinct differences in miRNA activity in infections with viable and HK BCG. As there was a difference in LAMP1 signal between phagosomes containing HK and viable BCG, we hypothesized that these differences might be regulated by miRNAs that control phogosomal processing. The direction of present bottom up approach was from protein-coding genes toward finding miRNAs, which is an opposite method to classical studies, where identification of given miRNAs involves association of targets (Sharbati et al., 2011; Hoeke et al., 2013). This approach is versatile and can also be used for other pathways, however the possibilities of finding regulatory miRNAs can be enhanced by pre-defining experimental conditions as it was shown by selection of time points for RNA isolation. The approach is very flexible at each step, e.g., selection criteria for protein-coding genes and selection of miRmap scores.

Next step in our approach was to search for miRNAs that target selected genes. In the present study, after selecting 10 top candidate miRNAs, three of them (miR-3619-5p, miR-637, and miR-324-3p) turned out to be regulated upon infection. The approach has narrowed down the study of vast number of miRNAs, leading to straightforward identification of regulated miRNAs. However, the method has failed to distinguish between regulating miRNAs after phagocytosis of viable and HK BCG. The reason could be due to narrowed selection of proteins. Selection of an increased number of protein-coding genes in the beginning as well as inclusion of other closely related pathways to phagocytosis might have helped finding differences in regulated miRNAs between viable and HK BCG infection. Nevertheless, the presented approach helped to detect regulated miRNAs, which potentially target protein-coding genes involved in phagolysosomal trafficking and processing. On a different note, PMA is a known PKC activator, and PKC has been reported to interfere with autophagic signaling during mycobacterial infection of macrophages (Kumar et al., 2015). However, in our experiments PMA was only taken for differentiation and was removed 24 h before infection/treatment of THP-1 derived macrophages and therefore is not thought to influence the experiments.

Generally, down-regulation of miRNAs corresponds to up-regulation of respective targets. After *in silico* and RT-qPCR based analysis, increased and anti-correlated expression of two predicted targets CTSS and RAB11FIP4 was observed in same RNA samples. Both protein-coding genes (CTSS and RAB11FIP4) were predicted to be mutually targeted by miR-3619-5p and miR-637 with miRmap scores more than 90. Based on KEGG pathway database, these two proteins have functional involvement in lysosome and endocytosis pathways, respectively (Kanehisa and Goto, 2000). In present study, increased CTSS expression at 4 h post infection was observed and miR-3619-5p was down-regulated already at 15 min. Lagged regulation of the target CTSS is a characteristic of miRNA regulation. The lysosomal cysteine proteinase CTSS acts as a late event after fusion of autophagosomes and lysosomes. Therefore, regulation of CTSS at 4 h post infection is consistent with the co-localization of LAMP1 signals as a late marker of phagolysosomal processing. In an earlier study, CTSS was up-regulated when mica fine particles were phagocytized by murine macrophage cell line, RAW 264.7 (Jung et al., 2015). Interestingly, over-expression of CTSS was found to be responsible for the regulation of autophagy and apoptosis (Chen et al., 2012). After transfection of THP-1 derived macrophages with miR-3619-5p and miR-637 mimics, down-regulation of CTSS but not that of RAB11FIP4 was observed only in miR-3619-5p mimics transfected cells. Down-regulation of CTSS by miR-3619-5p mimic was around 20%. In reporter gene assays, also 20% decreased luciferase activity was observed in miR-3619-5p transfected cells corresponding well to the moderately decreased cellular levels of CTSS transcripts. In an earlier study, after transfecting human cancer cell lines HONE 1 with CTSS siRNA, LC3B-II protein was accumulated as determined by western blots (Chen et al., 2012). Other recent studies also have suggested that inhibition of CTSS induces autophagy in different cells (Chen et al., 2012; Zhang et al., 2014; Huang et al., 2015). Based on KEGG pathway database, CTSS is involved in 4 different pathways (lysosome, phagosome, antigen processing and presentation, and tuberculosis). It is known that CTSS is involved in antigen processing and presentation (Riese et al., 1998; Driessen et al., 1999; Saegusa et al., 2002; Beers et al., 2005). So, in both infection groups (viable and HK), up-regulation of CTSS might be linked to degradation of phagocytized particles and antigen presentation. In another study, down-regulation of CTSS along with LAMP1 and Cathepsin D (CTSD) caused reduction in autophagosome-lysosome fusion (Burgos-Portugal et al., 2014). CTSS deficient mouse hearts were associated with abnormal accumulation of autophagosomes. As CTSS in lysosomes was essential for mitophagy processing in macrophages, its deficiency increased the damage to mitochondria and elevated ROS levels (Pan et al., 2012). In our experiments, miR-3619-5p as well as siRNA mediated CTSS knockdown resulted in increased number of LC3 puncta. Western blots showed increased cellular LC3-II levels but there was no reduction of p62 levels. p62 was even accumulated after specific knockdown of CTSS pointing to a block of lysosomal processing. This points out that the increased number of LC3 puncta as well as conversion might rather be grounded in blockage of lysosomal trafficking after CTSS down-regulation than induction of autophagy. Decreased levels of the lysosomal cysteine proteinase CTSS is likely to affect degradation of autophagosomal contents. In a recent publication it has been shown that Atg5 but not other authophagy genes plays a key role in the host response to mycobacteria (Behar and Baehrecke, 2015; Kimmey et al., 2015). On a different note, lysosome activation induced by mTOR inhibition has been shown to depend on Atg5/7, suggesting that lysosomal activation depends upon autophagosome formation. The authors have shown that activity of the lysosomal proteinases cathepsin B and L depends upon Atg5/7 mediated autophagosome-lysosome fusion, meaning that cathepsins act downstream of Atg5 (Zhou et al., 2013). Our study suggests that miR-3619-5p mediated down-regulation of CTSS impairs degradation of autophagic substrates thus blocking autophagosome-lysosome processing. This results rather in accumulation of autophagic bodies than induction of autophagy, which is consistent with recent observations of Kimmey et al. since Atg5 is also needed for lysosomal cathepsin activation.

In conclusion, the presented bottom up approach was successful in finding regulated miRNAs and targets, which have functions in phagocytosis related pathways. Besides its potential role in other pathways, down-regulation of miR-3619-5p and consequently accumulated CTSS seems to be a new regulatory axis relevant for the innate immune response to pathogenic mycobacteria.

## Author contributions

KP: Performed experiments, analyzed data and wrote manuscript; JS: Performed microscopy; RE: Contributed reagents, materials, analysis tools and writing; SS: Conceived the study, designed experiments, analyzed data, wrote manuscript and contributed reagents, materials and analysis tools.

## Funding

The study was funded by the German Academic Exchange Service (DAAD) and was supported by the German Research Foundation (DFG) through SFB 852 Project B4.

### Conflict of interest statement

The authors declare that the research was conducted in the absence of any commercial or financial relationships that could be construed as a potential conflict of interest.
