# Supplementary material for: Mycobacterium bovis BCG Interferes with miR-3619-5p Control of Cathepsin S in the Process of Autophagy
Source: Front Cell Infect Microbiol. 2016 Mar 9;6:27. doi: 10.3389/fcimb.2016.00027 (PMC4783571; doi:10.3389/fcimb.2016.00027)
Supplement: Supplementary file 1 [file Presentation1.PDF]

**Table 1: Name of proteins and their genes selected for *in silico* analysis.**

| Protein | Genes | KEGG Pathway | Reference |
|---|---|---|---|
| VATPase | ATP6V1A | Phagosome | (Singh et al., 2006) |
| LAMP | LAMP1 | Phagosome | (Kelley and Schorey 2003) |
| PIKFYVE | PIKFYVE | Phagosome | (Hazeki et al., 2012) |
| RILP | RILP | Phagosome | (Sun et al., 2007) |
| DYNC1H | DYNC1H1 | Phagosome | From KEGG pathway |
| STX7 | STX7 | Phagosome | (Sun-Wada et al., 2009) |
| SK-1 | SPHK2, SPHK1 | Phagosome | (Prakash et al., 2010) |
| CALM | CALML6, CALML5, CALML3 CALM1, CALM2, CALM3 | Phagosome | (Tebar et al., 1999) |
| CaN | PPP3CA, CALN | Tuberculosis | From KEGG pathway |
| Coronin 1 | CORO1A | Tuberculosis | (Schuller et al., 2001) |
| VPS34 | PIK3C3 | Tuberculosis | (Vergne et al., 2003) |
| CAMK II | CAMK2B, CAMK2D, CAMK2G | Tuberculosis | (Koul et al., 2004) |
| EEA1 | EEA1 | Lysosome | (Fratti et al., 2001) |
| MCOLN1 | MCOLN1 | Lysosome | (Pryor et al., 2006) |
| LIMP | CD63 | Lysosome | (Welin et al., 2011) |

**Table 2: Primer sequences of selected miRNAs and mRNAs**

| Nr | Name | RT6-miRNA | miRNA-rev |
|---|---|---|---|
| 1 | miR-4739 | tgtcaggcaaccgtattcaccgtgagtggt**aggcc** | cgtcagatgtccgagtagagggggaacggcg**aagggaggaggagcggaggggc** |
| 2 | miR-637 | tgtcaggcaaccgtattcaccgtgagtggt**acgcag** | cgtcagatgtccgagtagagggggaacggcg**actgggggctttcgggctctg** |
| 3 | miR-3619-5p | tgtcaggcaaccgtattcaccgtgagtggt**gctgca** | cgtcagatgtccgagtagagggggaacggcg**tcagcaggcaggctggtgc** |
| 4 | miR-324-3p | tgtcaggcaaccgtattcaccgtgagtggt**ccagca** | cgtcagatgtccgagtagagggggaacggcg**actgccccaggtgctgc** |
| 5 | miR-4446-5p | tgtcaggcaaccgtattcaccgtgagtggt**gccaag** | cgtcagatgtccgagtagagggggaacggcg**atttccctgccattccctt** |
| 6 | miR-4690-5p | tgtcaggcaaccgtattcaccgtgagtggt**ttcagc** | cgtcagatgtccgagtagagggggaacggcg**gagcaggcgaggctgggct** |
| 7 | miR-4709-3p | tgtcaggcaaccgtattcaccgtgagtggt**gctaca** | cgtcagatgtccgagtagagggggaacggcg**ttgaagaggaggtgctctgt** |
| 8 | miR-5006-5p | tgtcaggcaaccgtattcaccgtgagtggt**ttccac** | cgtcagatgtccgagtagagggggaacggcg**ttgccagggcaggaggtg** |
| 9 | miR-654-5p | tgtcaggcaaccgtattcaccgtgagtggt**gcacat** | cgtcagatgtccgagtagagggggaacggcg**tggtgggccgcagaacatg** |
| 10 | miR-761 | tgtcaggcaaccgtattcaccgtgagtggt**tgtgtc** | Cgtcagatgtccgagtagagggggaacggcg**gcagcagggtgaaactgac** |

**mRNAs**

| Nr | Name | Forward | Reverse | Product size |
|---|---|---|---|---|
| 1 | RAB11FIP4 | ggcgaggaggtggaaaaact | accgacagcacatccttcag | 131 |
| 2 | PIP4K2B | cgtgtgccagaaagtgaagc | gctgggcaggttctccttatt | 190 |
| 3 | RAB11FIP1 | gccaagcacagacttcatcct | tatgcaaatgcagggtccga | 152 |
| 4 | ZFYVE20 | gaccctccaccacatccaag | cagggcagcttgtctctcc | 186 |
| 5 | TRAF6 | aactgtgctgcatcaatggc | atgtgcatggaattggggct | 160 |
| 6 | CTSS | tggatcaccactggcatctc | accacaagaacccatgtctcc | 191 |

**Oligonucleotide sequence from three different region of CTSS**

| Nr | Name | CTSS_ts_sense | CTSS_ts_antisense |
|---|---|---|---|
| 1 | CTSS_ts | ggccgctatgaagcactttctcttaacttaattttt cctgctgtatccatgtttttgagacggagtctca ctctgttgcccagggtgactctgaccttctgcct gctgttctcct | ctagaggagaacagcaggcagaaggtcagagt caccctgggcaacagagtgagactccgtctcaaa aaacatggatacagcaggaaaaattaagttaaga gaaagtgcttcatagc |

## Figure 1

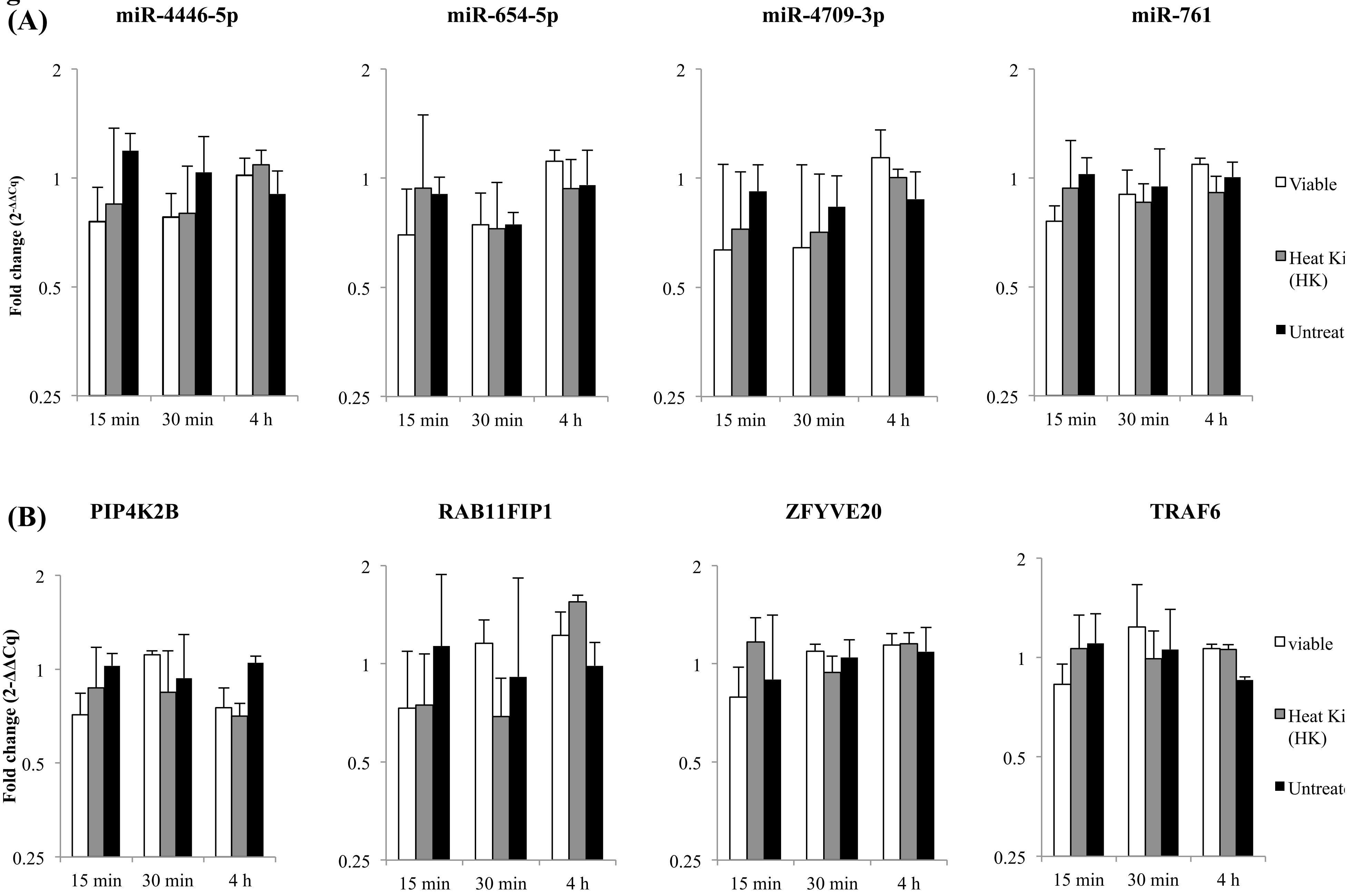


Expression of miRNAs and their predicted targets at 15 min 30 min and 4 h in viable and heat killed (HK) mycobacterial infection to macrophages. (A) The miRNAs (miR-4446-5p, miR-654-5p, miR-4709-3p and miR-761) and (B) their predicted targets (PIP4K2B, RAB11FIP1, ZFYVE20 and TRAF6) were not regulated in all time points.

**Figure 2**

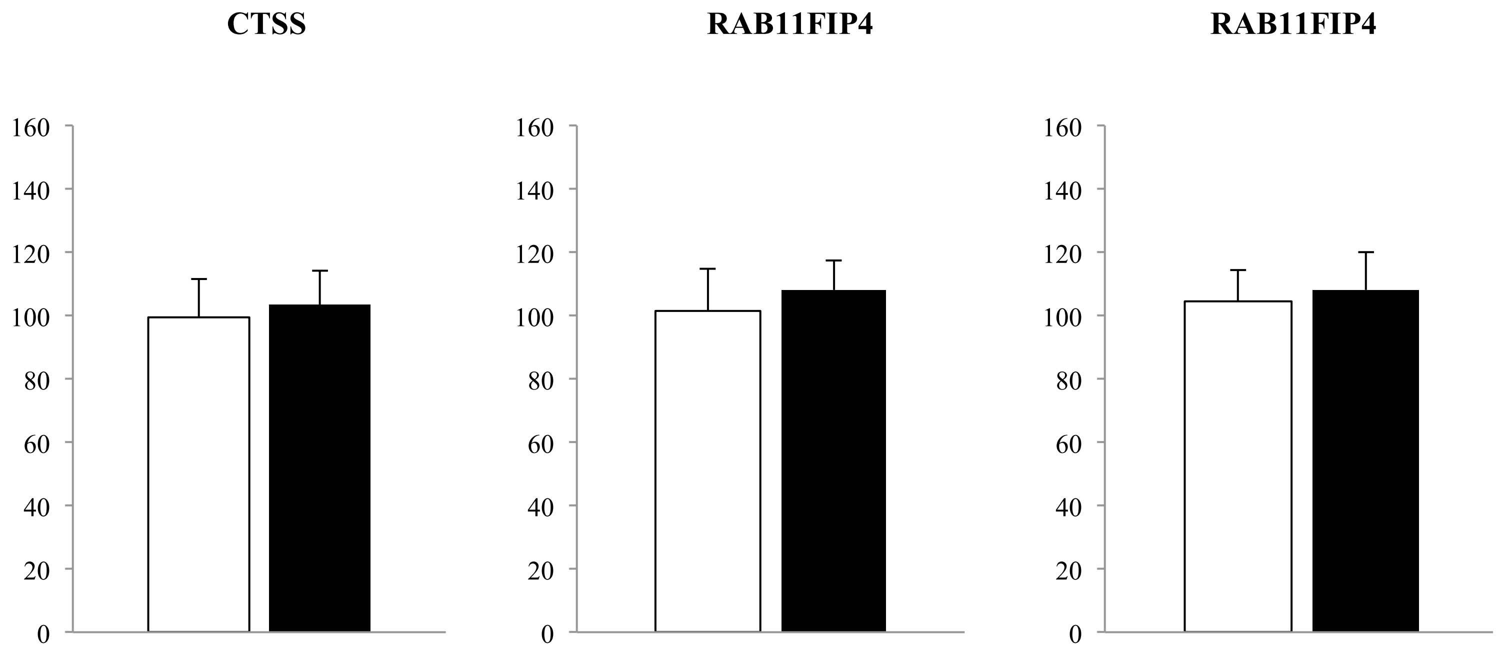


| | | | | | | |
|---|---|---|---|---|---|---|
| miR-3619-5p | - | - | + | - | - | - |
| miR-637 | + | - | - | - | + | - |
| nonsense control | - | + | - | + | - | + |

Expression of CTSS and RAB11FIP4 in THP-1 cells transfected with miRNAs mimics (either hsa-miR-637 or hsa-miR-3619-5p). No regulation was observed for CTSS after transfecting cells with hsa-miR-637. Also, no regulation was observed for RAB11FIP4 after transfecting cells either with miR-637 or miR-3619-5p mimics.

**Reference for table 1**

Fratti R. A., Backer J. M., Gruenberg J., Corvera S. and Deretic V. (2001). Role of phosphatidylinositol 3-kinase and Rab5 effectors in phagosomal biogenesis and mycobacterial phagosome maturation arrest. *J Cell Biol.* **154**:3. doi: 10.1083/jcb.200106049

Hazeki K., Nigorikawa K., Takaba Y., Segawa T., Nukuda A., Masuda A., et al. (2012). Essential roles of PIKfyve and PTEN on phagosomal phosphatidylinositol 3-phosphate dynamics. *FEBS Lett.* **586**:22. doi: 10.1016/j.febslet.2012.09.043

Kelley V. A. and Schorey J. S. (2003). Mycobacterium's arrest of phagosome maturation in macrophages requires Rab5 activity and accessibility to iron. *Mol Biol Cell.* **14**:8. doi: 10.1091/mbc.E02-12-0780

Koul A., Herget T., Klebl B. and Ullrich A. (2004). Interplay between mycobacteria and host signalling pathways. *Nature Reviews Microbiology.* **2**:3. doi: 10.1038/nrmicro840

Prakash H., Luth A., Grinkina N., Holzer D., Wadgaonkar R., Gonzalez A. P., et al. (2010). Sphingosine kinase-1 (SphK-1) regulates Mycobacterium smegmatis infection in macrophages. *PLoS One.* **5**:5. doi: 10.1371/journal.pone.0010657

Pryor P. R., Reimann F., Gribble F. M. and Luzio J. P. (2006). Mucolipin-1 is a lysosomal membrane protein required for intracellular lactosylceramide traffic. *Traffic.* **7**:10. doi: 10.1111/j.1600-0854.2006.00475.x

Schuller S., Neefjes J., Ottenhoff T., Thole J. and Young D. (2001). Coronin is involved in uptake of Mycobacterium bovis BCG in human macrophages but not in phagosome maintenance. *Cellular Microbiology.* **3**:12. doi: DOI 10.1046/j.1462-5822.2001.00155.x

Singh C. R., Moulton R. A., Armitige L. Y., Bidani A., Snuggs M., Dhandayuthapani S., et al. (2006). Processing and presentation of a mycobacterial antigen 85B epitope by murine macrophages is dependent on the phagosomal acquisition of vacuolar proton ATPase and in situ activation of cathepsin D. *J Immunol.* **177**:5.

Sun J., Deghmane A. E., Soualhine H., Hong T., Bucci C., Solodkin A., et al. (2007). Mycobacterium bovis BCG disrupts the interaction of Rab7 with RILP contributing to inhibition of phagosome maturation. *J Leukoc Biol.* **82**:6. doi: 10.1189/jlb.10.1189

Sun-Wada G. H., Tabata H., Kawamura N., Aoyama M. and Wada Y. (2009). Direct recruitment of H+-ATPase from lysosomes for phagosomal acidification. *J Cell Sci.* **122**:Pt 14. doi: 10.1242/jcs.050443

Tebar F., Bohlander S. K. and Sorkin A. (1999). Clathrin assembly lymphoid myeloid leukemia (CALM) protein: localization in endocytic-coated pits, interactions with clathrin, and the impact of overexpression on clathrin-mediated traffic. *Mol Biol Cell.* **10**:8.

Vergne I., Chua J. and Deretic V. (2003). Tuberculosis toxin blocking phagosome maturation inhibits a novel Ca2+/calmodulin-PI3K hVPS34 cascade. *J Exp Med.* **198**:4. doi: 10.1084/jem.20030527

Welin A., Raffetseder J., Eklund D., Stendahl O. and Lerm M. (2011). Importance of phagosomal functionality for growth restriction of Mycobacterium tuberculosis in primary human macrophages. *J Innate Immun.* **3**:5. doi: 10.1159/000325297
